# Asymptomatic hyperuricemia associated with increased risk of nephrolithiasis: a cross-sectional study

**DOI:** 10.1186/s12889-023-16469-y

**Published:** 2023-08-10

**Authors:** Haoyuan Deng, Xuehui Zhang, Nan Cheng, Jianghui Zhang, Chongwei Song, Yunrui Sun, Zhongxin Hou, Yi Li, Qian Wang, Jianzhong Yin, Qiong Meng

**Affiliations:** 1 School of Public Health, Kunming Medical University, 1168 West Chunrong Road, Yuhua Avenue, Chenggong District, Kunming, 650500 Yunnan China; 2 Present Address: The First Affiliated Hospital, Kunming Medical University, 295 Xichang Road, Kunming, 650032 Yunnan China; 3 Present Address: Department of Public Health, Wuhan Mental Health Center, Gongnongbing Road, Jiangan District, Wuhan, 430014 Hubei China; 4 AIDS Care Center, Yunnan Provincial Hospital of Infectious Disease, Anning District, Kunming, 650399 Yunnan China; 5 Present Address: Baoshan College of Traditional Chinese Medicine, Baoshan Longyang District Qingyang District Vocational Education Park, Baoshan, 678000 Yunnan China

**Keywords:** Nephrolithiasis, Serum uric acid, Cross-sectional study, Restricted cubic splines

## Abstract

**Background:**

Existing evidence shows that there is an independent correlation between nephrolithiasis and gout, and hyperuricemia is the most important risk factor for gout. However, hyperuricemia was often used as an accompanying symptom of gout to explore its association with nephrolithiasis, there were few studies to explore whether hyperuricemia itself or serum uric acid (SUA) is related to the risk of nephrolithiasis. Evidence on the relationship between hyperuricemia and nephrolithiasis is still insufficient.

**Methods:**

A total of 22,303 participants aged 30 to 79 years who participated in the China Multi-Ethnic Cohort (CMEC) study in Yunnan Province from May 2018 to September 2019 were included in the study. All participants received standardized face-to-face interviews, medical examinations, and biochemical examinations. Logistic regression was used to estimate the association between hyperuricemia and nephrolithiasis, and a restricted cubic spline (RCS) model was used to explore the dose–response relationship between SUA and the risk of nephrolithiasis.

**Results:**

14.5% of all participants were diagnosed with hyperuricemia, and 12.1% were diagnosed with nephrolithiasis. After adjusting for all potential confounders, the OR (95%CI) for nephrolithiasis in participants with hyperuricemia compared with participants without hyperuricemia was 1.464 (1.312,1.633), *p* < 0.001. Restricted cubic spline regression analysis showed that the risk of nephrolithiasis increased with the increase of SUA, and when the level of SUA is higher than 356 μmol/L in males and higher than 265 μmol/L in females, there is a dose–response relationship between the increase of SUA and the risk of nephrolithiasis in both males and females (*p* for nonlinearity = 0.1668, *p* for nonlinearity = 0.0667).

**Conclusion:**

Asymptomatic hyperuricemia is associated with an increased risk of developing nephrolithiasis. Before reaching the diagnostic criteria for hyperuricemia, the risk of nephrolithiasis rises with the increase in SUA. This suggests that controlling SUA levels may be significant for the prevention of nephrolithiasis.

**Supplementary Information:**

The online version contains supplementary material available at 10.1186/s12889-023-16469-y.

## Introduction

In modern society, nephrolithiasis is highly prevalent all over the world. Previous studies have shown that the incidence of nephrolithiasis is 7–13% in North America, 5–9% in Europe, and 1–5% in Asia, and the global incidence and prevalence of nephrolithiasis continue to increase [1, 2]. In addition, nephrolithiasis is a very common clinical systemic disease that was associated with a significant increase in the likelihood of hypertension, chronic kidney disease, end-stage renal disease (ESRD), or other adverse renal outcomes [3–5]. It increases the risk of coronary artery disease and ischemic stroke and is associated with bone loss and fractures, type 2 diabetes, and metabolic syndrome [6, 7]. At the same time, nephrolithiasis is a recurrent disease, with a recurrence rate of 50% in 5–10 years and 75% in 20 years. Recurrent nephrolithiasis will significantly reduce the level of renal function and significantly increase the risk of chronic kidney disease (CKD) [8, 9]. The latest data show that by 2016, the prevalence of nephrolithiasis in China was about 5.8%-7.5% [10, 11]. China is the most populous country in the world, therefore, the number of kidney stone patients in China is very likely to be huge, which brings a heavy socio-economic and medical burden.

Existing evidence shows that the pathogenesis of nephrolithiasis is complex, and the occurrence of nephrolithiasis is associated with body fat content, dyslipidemia, and a high risk of cardiovascular events [12–14]. In addition, studies have shown that there is an independent correlation between nephrolithiasis and gout [15]. Hyperuricemia is the most important risk factor for gout [16], and some researchers even believe that hyperuricemia is defined as serum uric acid (SUA) above 360 µmol/L (6.0 mg/dL) because the lifetime risk of gout seems to start at this level. Therefore, it is very reasonable to believe that SUA levels play an important role in the independent association between nephrolithiasis and gout. However, hyperuricemia is often used as an accompanying symptom of gout to explore its association with nephrolithiasis. There are limited studies to explore whether hyperuricemia itself or SUA levels are related to the risk of nephrolithiasis, especially the large population studies that are rare. In this study, our main purpose was to use cross-sectional data from a multi-ethnic cohort study in Southwest China to explore the relationship between asymptomatic hyperuricemia and the risk of nephrolithiasis and to try to discover a reference value for SUA to prevent the development of nephrolithiasis.

## Materials and methods

### Study design and population

**Fig. 1:**
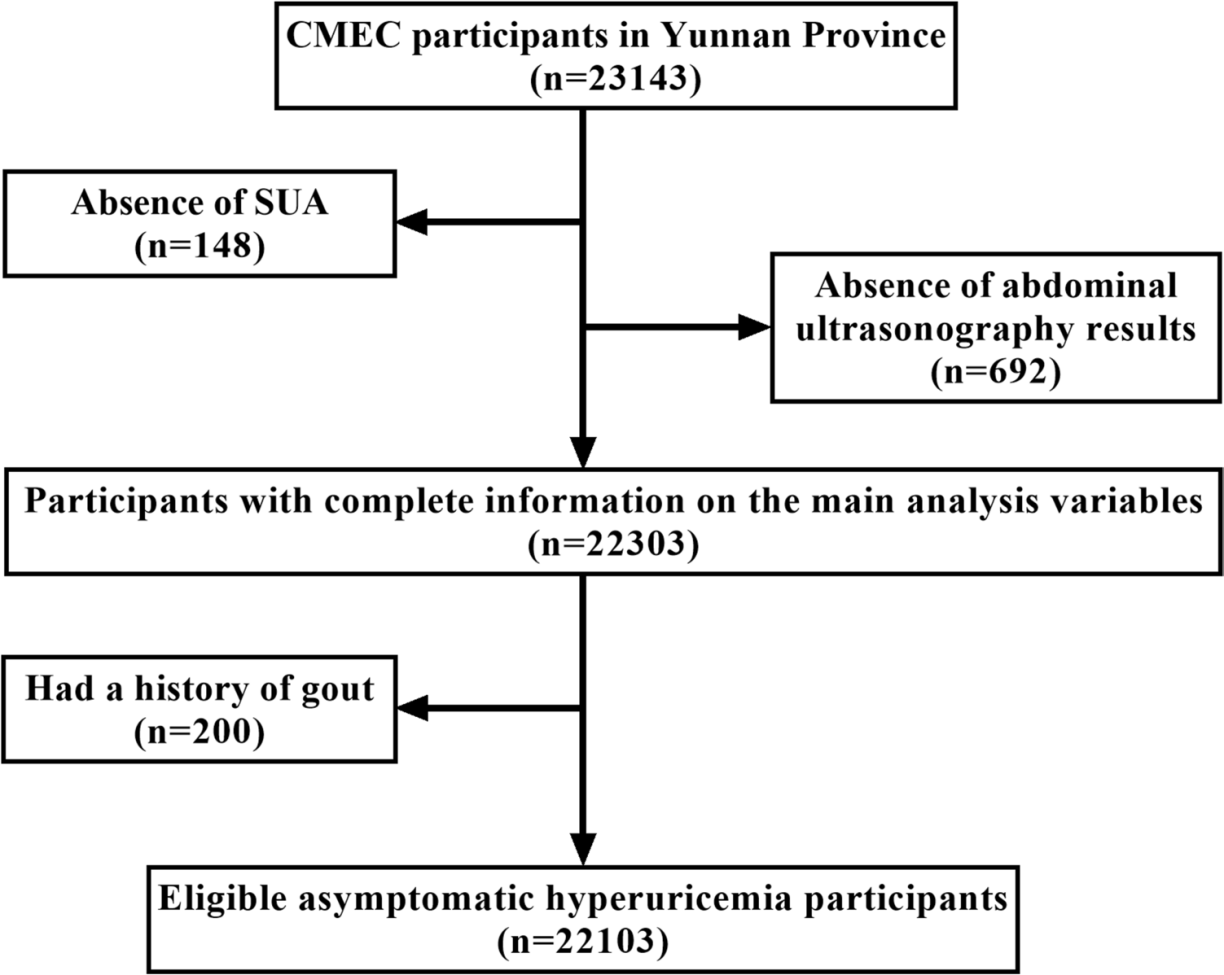
Flowchart of participants’ recruitment and study inclusion

We used the baseline data from the China Multi-Ethnic Cohort (CMEC) study. The CMEC study is a prospective cohort study that fully considered China's ethnic characteristics, population size, and non-communicable disease patterns. Five provinces (Chongqing, Guizhou, Sichuan, Tibet, and Yunnan) in southwestern China were selected for the survey study from May 2018 to September 2019, with participants ranging in age from 30 to 79 years old [17]. The sample in this study was obtained from community-based populations in Yunnan province using a multistage stratified cluster sampling method. In the first phase, four ethnic minority settlements (Wuding, Yongren, Heqing, and Yongsheng counties) were selected as our study sites, mainly including the Yi, Bai, and Han ethnic groups. In Yongsheng County, the Han ethnic group accounts for 65.07%. In Heqing County, the Bai ethnic group accounts for 59%. In Wuding County, the Yi ethnic group accounts for 32.08%. The Yi ethnic group of Yongren accounts for 55.5%. In the second phase, we selected 2-8 communities in each region based on community size. Finally, participants who met the inclusion-exclusion criteria were invited to participate in our study. Inclusion criteria: (a) age 30-79 years on the day of the survey; (b) being a resident of the survey site for generations and able to complete the baseline survey as well as the follow-up study; (c) being able to complete the questionnaire, physical examination, and blood tests. Exclusion criteria: (a) unable to provide a unique national ID card; (b) suffering from severe mental illness (e.g., schizophrenia and bipolar disorder); and (c) refusing to comply with the study requirements. According to the inclusion-exclusion criteria, a total of 23,143 participants were enrolled. The participants who did not have abdominal ultrasound results (*n*=692) or SUA levels(*n*=148) and those with a history of gout (*n*=200) were excluded.22,103 participants were included in our study finally (Fig. 1). All participants signed informed consent before data collection and this study was approved by Kunming Medical University Medical Ethical Review Board (KMMU2020MEC078).

### The implementation of investigation and physical examination

All participants were interviewed by some trained investigators using electronic questionnaires. Demographic characteristics, life behaviors, history of diseases (including hypertension, diabetes, and hyperlipidemia), and other information were collected through the questionnaires. In addition, fasting blood was drawn from all participants for biochemical tests. The values of Glycated hemoglobin (HbA1C), Fasting blood glucose (FBG), Cholesterol, Triglycerides (TG), high-density lipoprotein cholesterol (HDL-CH), etc. were obtained through laboratory tests of blood samples. Some physical examinations (height, weight, blood pressure) and abdominal B-ultrasound examination (including the liver and kidneys) were carried out after the questionnaires were completed. The blood pressure was measured (using OMRON HEM-8711) at least three times after the respondents rested for five minutes. The above examinations shall be completed by doctors who have obtained the qualification certificate of licensed doctors and received standardized training to ensure the accuracy of the data. The above measuring instruments have passed the inspection of the quality inspection department.

### Definition of nephrolithiasis and hyperuricemia

According to previously published studies related to cardiovascular outcomes, clinical laboratories typically use SUA concentration cutoffs of 420 µmol/L (7.0 mg/dL) for men and 360 µmol/L (6.0 mg/dL) for women to define hyperuricemia [18]. The diagnostic criteria for nephrolithiasis were: (a) diagnosed by abdominal ultrasonography, which was performed by specialist physicians; (b) had been diagnosed with nephrolithiasis by a doctor in a hospital at the township/district level or above. Nephrolithiasis was diagnosed when any of the above two criteria were met. The prevalence of nephrolithiasis mentioned in this study refers to the sum of the prevalence of nephrolithiasis obtained by abdominal ultrasound diagnosis and the cumulative incidence obtained by questionnaire survey.

### Assessment of covariates

Assessment of some covariates about life behaviors was as follows: (a) smoking status (including non-smoking, smoking, and quitting), where non-smokers were defined as never smokers or occasional smokers (the respondents who had smoked no more than 100 cigarettes in their lifetime as of the survey day); smokers were defined as those who had smoked more than 100 cigarettes cumulatively in their lifetime and less than six months of continuous cessation behavior; ex-smokers were defined as those who had smoked more than 100 cigarettes cumulatively but had at least six months of continuous cessation behavior. (b) drinking frequency: ask "How often did you drink alcohol in the past year", and "if you drink at least once a week, how many days a week did you drink alcohol in the past year", combine the two questions to define the frequency of drinking (never or almost no alcohol, occasional, 1–2 days/week, 3–5 days/week, daily). (c) tea drinking situation: ask "Whether you drink tea every week and last for more than half a year", and "if yes, how many days per week did you drink tea on average in the past year", to get the frequency of tea drinking (no drinking, 1–2 days /week, 3–5 days/week, drinking every day). (d) habits of napping: ask "Do you have a habit of napping?".

The diagnostic criteria for hypertension were: (a) systolic blood pressure ≥ 140 mmHg and/or diastolic blood pressure ≥ 90 mmHg; (b) have been diagnosed with hypertension by a doctor in a hospital at the township/district level or above. If the above criteria were met, the diagnosis was hypertension.

The diagnostic criteria for diabetes [19]: (a) FBG ≥ 7.0 mmol/L, fasting refers to no calorie intake for at least 8 h; (b) the last meal was less than 8 h after blood collection, random blood glucose (RBG) ≥ 11.1 mmol/L; (c) HbA1C ≥ 6.5%; (d) diagnosed with diabetes by a doctor in a hospital at the township/district level or above. Diabetes was diagnosed when any of the above criteria were met.

The diagnostic criteria for hyperlipidemia [20]: (a) hypercholesterolemia: cholesterol ≥ 6.2 mmol/L, TG < 2.3 mmol/L; (b) hypertriglyceridemia: cholesterol < 6.2 mmol/L, TG ≥ 2.3 mmol/L; (c) mixed hyperlipidemia: cholesterol ≥ 6.2 mmol/L, TG ≥ 2.3 mmol/L; (d) low-density lipoprotein cholesterolemia: HDL-CH < 1.0 mmol/L; (e) diagnosed with diabetes by a doctor in a hospital at the township/district level or above. Meeting the above criteria can be diagnosed as hyperlipidemia.

### Statistical analysis

In this study, to compare the distribution characteristics of individuals with and without nephrolithiasis, the mean (SD) was used to describe continuous variables and the number (percentage) for categorical variables. Univariate and multivariate binary logistic regression were used to screen and adjusted potential confounders and to explore the independent relationship between hyperuricemia and nephrolithiasis. Model 1 was a crude model without any adjustment. Demographic features, life behavior factors, and metabolic-related indicators and diseases were successively adjusted in model 2 to model 4. Additionally, stratified analysis was conducted to compare the relationship between nephrolithiasis and hyperuricemia in different age groups and gender groups and ethnic groups by using a fully adjusted model (Model 4). Restricted cubic splines with five knots were used to flexibly model the linear relationship between SUA and the prevalence of nephrolithiasis which was stratified by sex. The restricted cubic spline models were adjusted for the covariates mentioned above (Model 4).

To assess the robustness of our findings, we also performed a sensitivity analysis. We excluded patients with self-reported nephrolithiasis and redefines nephrolithiasis patients as subjects diagnosed with nephrolithiasis by abdominal ultrasonography during the field survey. All *p* values were two-sided, and *p* < 0.05 was considered statistically significant. SPSS software (version 27.0) and/or R software (version 4.1.2) were used for the data analyses.

## Results

A total of 22,103 participants between the ages of 30 and 79 were included in our analysis. The mean age of study participants was 52.9 ± 10.4 years, most of them were female (67.69%), and mainly from rural areas (92.96%). The participants were of Han nationality, Bai nationality, and Yi nationality, accounting for 45.51%, 26.82% and 27.67% respectively.92.6% of them had a junior high school education or below (Table 1). There were more women than men in our study population because our study site is located in a rural community in Yunnan, where many young and middle-aged men choose to work outside the community to earn household income.

**Table 1:** Basic characteristics of the participants

Variables	Group	Male (*n* = 7141)	Female (*n* = 14962)	All participants (*n* = 22103)
Age, years	≤ 44	1316(18.43)	3419(22.85)	4735(21.42)
	45–59	3579(50.12)	7783(52.02)	11362(51.40)
	≥ 60	2246(31.45)	3760(25.13)	6006(27.17)
Ethnic Groups	Han nationality	3462(48.48)	6597(44.09)	10059(45.51)
	Yi nationality	1915(26.82)	4014(26.83)	5929(26.82)
	Bai nationality	1764(24.70)	4351(29.08)	6115(27.67)
Household registration	Rural	6462(90.49)	14056(93.94)	20518(92.96)
	Urban	579(8.11)	736(4.92)	1315(5.96)
	Unified^a^	89(1.25)	150(1.00)	239(1.08)
Annual household income, ¥	< 1200	1460(20.45)	3138(20.97)	4598(20.83)
	12000–19999	1527(21.38)	3655(24.43)	5182(23.48)
	20000–59999	2979(41.72)	6206(41.48)	9185(41.62)
	60000–99999	676(9.47)	1110(7.42)	1786(8.09)
	100000–199999	380(5.32)	677(4.52)	1057(4.79)
	≥ 200000	113(1.58)	149(1.00)	262(1.19)
Educational level	No formal	781(10.94)	5691(38.04)	6472(29.28)
	Primary school	2884(40.39)	5756(38.47)	8640(39.09)
	Junior high school	2674(37.45)	2704(18.07)	5378(24.33)
	high school	566(7.93)	487(3.25)	1053(4.76)
	College or above	236(3.3)	323(2.16)	559(2.53)

^a^The unified resident household registration that does not distinguish between urban and rural household registration

In the total population, 2678(12.1%) participants were diagnosed with nephrolithiasis. The prevalence of nephrolithiasis was higher in men (15.71%) than in women (10.40%), and the prevalence of nephrolithiasis in Bai nationality (13.4%) and Yi nationality (15.2%) was higher than that of Han nationality (9.5%). The prevalence of nephrolithiasis in middle-aged people aged 45 to 59 was higher than that in young people under 45 and older people over 60. Compared to the characteristics of participants who did not have nephrolithiasis, those who had nephrolithiasis tended to be current or ex-smokers, to have high body fat, suffer from hypertension, diabetes, fatty liver, or hyperlipidemia (Table 2).

**Table 2:** The prevalence of nephrolithiasis in people with different characteristics and the results of univariate binary logistic regression analysis

Variables	Group	n(N)	Nephrolithiasis		OR (95%CI)
			Prevalence	95%CI	
Age, years	≤ 44	530(4735)	11.19	(10.29,12.09)	Ref
	45–59	1445(11362)	12.72	(12.11,13.33)	1.156(1.040,1.285)
	≥ 60	703(6006)	11.70	(10.89,12.51)	1.052(0.933,1.186)
Gender	Male	1122(7141)	15.71	(14.87,16.55)	Ref
	Female	1556(14962)	10.40	(9.91,10.89)	0.623(0.573,0.676)
Ethnic Groups	Han nationality	957(10,059)	9.51	(8.94,10.08)	Ref
	Yi nationality	902(5929)	15.21	(14.3,16.12)	1.707(1.548,1.881)
	Bai nationality	819(6115)	13.39	(12.54,14.24)	1.471(1.332,1.624)
Household registration	Rural	2481(20518)	12.09	(11.64,12.54)	Ref
	Urban	167(1315)	12.70	(10.90,14.50)	1.058(0.894,1.251)
	Unified	24(239)	10.04	(6.23,13.85)	0.812(0.531,1.240)
Annual household income, ¥	< 1200	581(4598)	12.64	(11.68,13.60)	Ref
	12000–19999	611(5182)	11.79	(10.91,12.67)	0.924(0.819,1.043)
	20000–59999	1096(9185)	11.93	(11.27,12.59)	0.937(0.841,1.043)
	60000–99999	219(1786)	12.26	(10.74,13.78)	0.966(0.818,1.141)
	100000–199999	122(1057)	11.54	(9.61,13.47)	0.902(0.733,1.110)
	≥ 200000	41(262)	15.65	(11.25,20.05)	1.283(0.909,1.810)
Educational level	No formal	739(6472)	11.42	(10.65,12.19)	Ref
	Primary school	1047(8640)	12.12	(11.43,12.81)	1.070(0.968,1.182)
	Junior high school	696(5378)	12.94	(12.04,13.84)	1.153(1.033,1.288)
	High school	126(1053)	11.97	(10.01,13.93)	1.054(0.862,1.290)
	College or above	70(559)	12.52	(9.78,15.26)	1.111(0.855,1.443)
Smoking status	Never	1874(16951)	11.06	(10.59,11.53)	Ref
	Current	690(4478)	15.41	(14.35,16.47)	1.465(1.334,1.610)
	Former	114(672)	16.96	(14.12,19.8)	1.644(1.336,2.022)
Drinking frequency	Never	1898(16378)	11.59	(11.10,12.08)	Ref
	Occasionally	482(3363)	14.33	(13.15,15.51)	1.276(1.146,1.421)
	1–2 days/week	33(290)	11.38	(7.72,15.04)	0.980(0.680,1.412)
	3–5 days/week	40(247)	16.19	(11.60,20.78)	1.474(1.047,2.075)
	Everyday	223(1812)	12.31	(10.80,13.82)	1.071(0.923,1.242)
Tea Drinking status	Never	1791(14867)	12.05	(11.53,12.57)	Ref
	1–2 days/week	59(679)	8.69	(6.57,10.81)	0.695(0.530,0.912)
	3–5 days/week	66(490)	13.47	(10.45,16.49)	1.136(0.873,1.480)
	Everyday	746(5980)	12.47	(11.63,13.31)	1.041(0.950,1.140)
Habit of napping	No	2033(16671)	12.19	(11.69,12.69)	Ref
	Yes	609(5211)	11.69	(10.82,12.56)	0.953(0.865,1.049)
BMI, kg/m^2^	< 18.5	162(1552)	10.44	(8.92,11.96)	Ref
	18.5–24.9	1765(14731)	11.98	(11.46,12.50)	1.168(0.985,1.385)
	≥ 25	712(5565)	12.79	(11.91,13.67)	1.259(1.051,1.508)
FBG, mg/dL	-	-	-	-	1.049(1.020,1.078)
Hypertension	No	1619(14688)	11.02	(10.51,11.53)	Ref
	Yes	1059(7415)	14.28	(13.48,15.08)	1.345(1.238,1.461)
Diabetes	No	2402(20112)	11.94	(11.49,12.39)	Ref
	Yes	276(1991)	13.86	(12.34,15.38)	1.187(1.038,1.357)
Hyperlipidemia	No	1680(14869)	11.30	(10.79,11.81)	Ref
	Yes	998(7234)	13.80	(13.01,14.59)	1.256(1.155,1.366)
Fatty Liver	No	2091(17751)	11.78	(11.31,12.25)	Ref
	Yes	587(4352)	13.49	(12.48,14.50)	1.168(1.058,1.288)
Hyperuricemia	No	2139(18908)	11.31	(10.86,11.76)	Ref
	Yes	539(3195)	16.87	(15.57,18.17)	1.591(1.435,1.763)
Scr^a^, µmol/L	-	80.50 ± 22.59	-	-	1.010(0.997,1.023)
Urea^a^, mmol/L	-	5.27 ± 1.58	-	-	1.036(0.983,1.089)

^a^Serum creatinine and urea are numerical variables and are described as $\overline{x }\pm s$; Scr: Serum Creatinine

To investigate the relationship between hyperuricemia and nephrolithiasis, we first performed a multivariate logistic regression analysis with hyperuricemia as the only independent variable, and the results showed a significant positive correlation between the two, and hyperuricemia was a risk factor for nephrolithiasis, the OR (95%CI) was 1.592, (1.436,1.764). Then we sequentially adjusted for participants' demographic characteristics, lifestyle behavior factors, and metabolism-related indicators and diseases, and found that hyperuricemia was still a risk factor for nephrolithiasis, the ORs with 95%CIs were 1.555(1.398,1.730), 1.559(1.401,1.735), 1.464(1.312,1.633). In addition, based on Model 4, we found that men had a higher risk of nephrolithiasis compared with women, and Yi and Bai’s people had a higher risk of nephrolithiasis than Han people; hypertension and hyperlipidemia were risk factors for nephrolithiasis, and daily alcohol consumption was a protective factor for nephrolithiasis compared to people who never drank alcohol (Table 3).

**Table 3:** The association between hyperuricemia and nephrolithiasis based on the multivariate binary logistic regression

Covariate	OR (95%CI)			
	Model 1	Model 2	Model 3	Model 4
Hyperuricemia				
No	Ref	Ref	Ref	Ref
Yes	1.592(1.436,1.764)***	1.555(1.398,1.730)***	1.559(1.401,1.735)***	1.464(1.312,1.633)***
Sex				
Male		Ref	Ref	Ref
Female		0.648(0.595,0.705)***	0.611(0.554,0.674)***	0.614(0.557,0.678)***
Ethnic Groups				
Han nationality		Ref	Ref	Ref
Yi nationality		1.790(1.623,1.975)***	1.775(1.609,1.959)***	1.725(1.562,1.905)***
Bai nationality		1.583(1.431,1.750)***	1.562(1.411,1.729)***	1.540(1.391,1.705)***
Drinking frequency				
Never			Ref	Ref
Occasionally			1.006(0.894,1.132)	1.018(0.904,1.146)
1–2 days/week			0.723(0.498,1.050)	0.721(0.496,1.047)
3–5 days/week			1.020(0.717,1.449)	1.008(0.708,1.433)
Everyday			0.766(0.651,0.903)**	0.760(0.645,0.896)**
Hypertension				
No				Ref
Yes				1.207(1.107,1.316)***
Hyperlipidemia				
No				Ref
Yes				1.110(1.017,1.212)*

Bolden numbers indicate statistical significance (*p < 0.05, **p < 0.01, ***p < 0.001)

Model 1: crude model (without adjustment)

Model 2: Model 1 adjusted for demographic features (i.e., age, sex, ethnic group, educational level)

Model 3: Model 2 adjusted for life behavior factors (i.e., smoking status, drinking frequency)

Model 4: Model 3 adjusted for metabolic-related indicators and diseases (i.e., BMI, fasting blood glucose, Scr, Urea, hypertension, diabetes, fatty liver, hyperlipidemia)

**Fig. 2:**
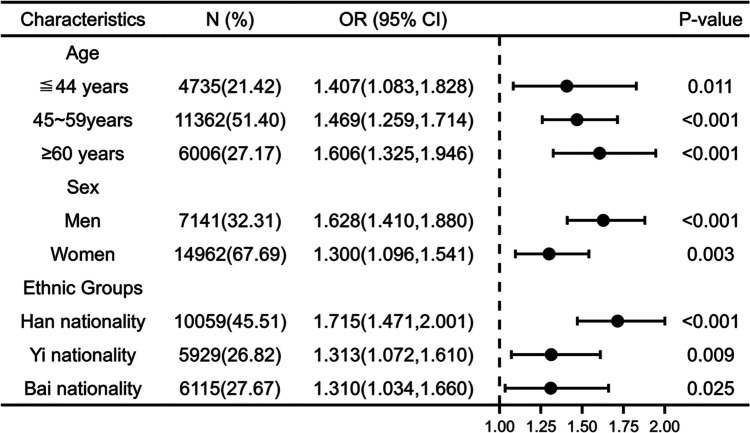
The association between hyperuricemia and nephrolithiasis after stratifying by age, gender, and ethnic group*. Note:* Adjusted for demographic features (i.e., age, sex, ethnic groups, educational level), behavioral factors (i.e., smoking status, drinking frequency), and metabolic-related indicators and diseases (i.e., BMI, fasting blood glucose, Scr, Urea, hypertension, diabetes, fatty liver, hyperlipidemia)

**Fig. 3:**
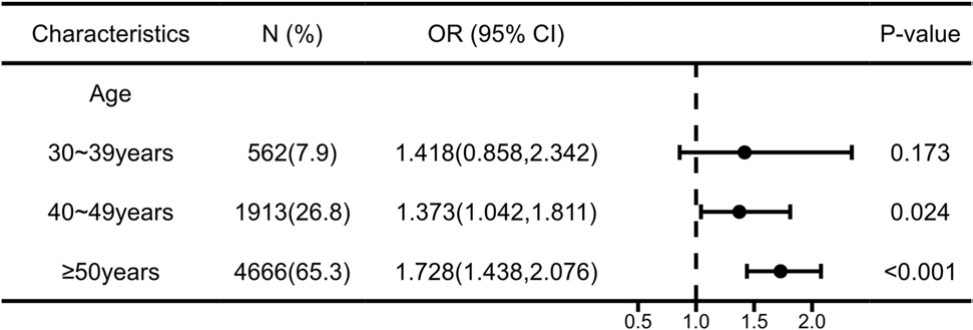
The association between hyperuricemia and nephrolithiasis after stratifying by age in females. *Note:* Adjusted for demographic features (i.e., ethnic groups, educational level), behavioral factors (i.e., smoking status, drinking frequency), and metabolic-related indicators and diseases (i.e., BMI, fasting blood glucose, Scr, Urea, hypertension, diabetes, fatty liver, hyperlipidemia)

After stratifying analyses for different genders, different ages, and different ethnic groups, we found that hyperuricemia remained positively associated with nephrolithiasis in each group (see Fig. 2 for details). Women were further stratified by age, with an average age of menopause of 50 years as an important node. The results showed that nephrolithiasis was still significantly associated with hyperuricemia in the 40–49 age group and in those older than 50 years. And the association between hyperuricemia and nephrolithiasis appears to be stronger in the age group older than 50 years (Fig. 3).

### Dose–response relationship between SUA and nephrolithiasis

**Fig. 4:**
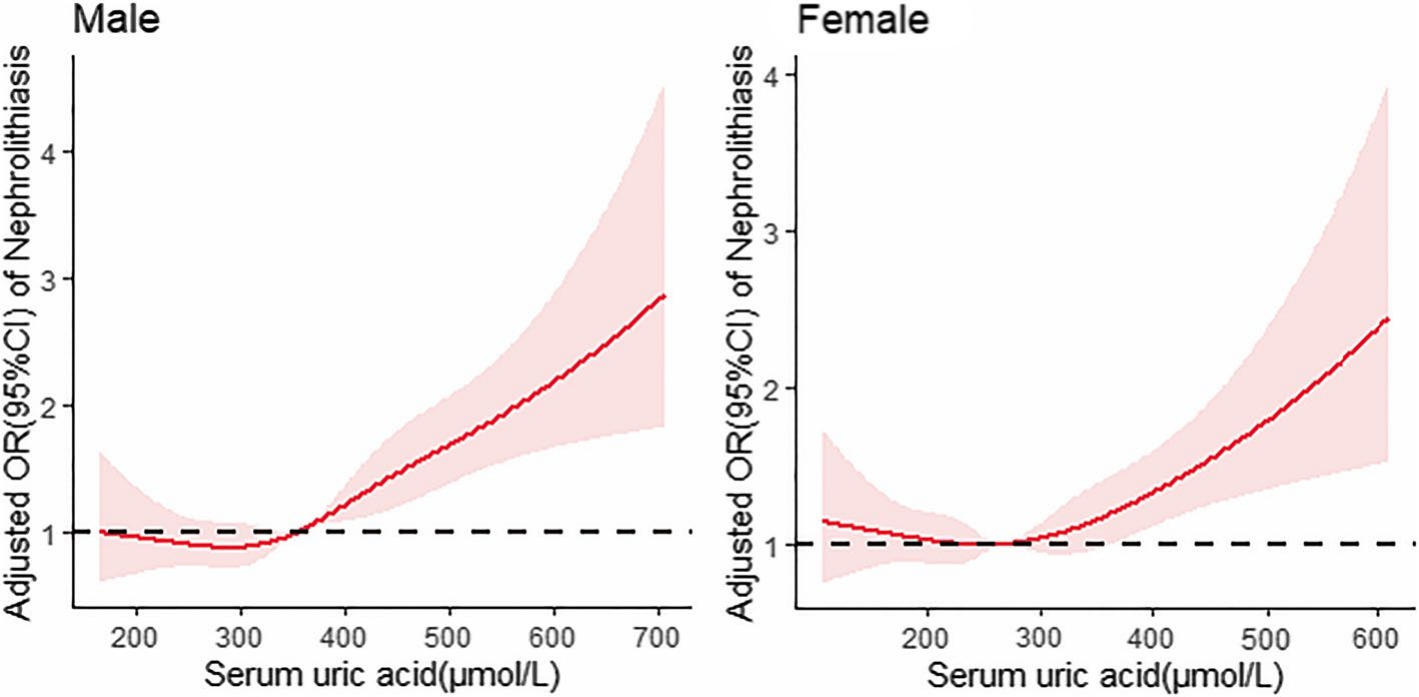
Dose–response relationship between SUA and nephrolithiasis in males and females. *Note:* Adjusted for age, sex, ethnic groups, educational level, smoking status, drinking frequency, BMI, fasting blood glucose, Scr, Urea, hypertension, diabetes, fatty liver, and hyperlipidemia

Restricted cubic spline models showed a linear dose–response relationship between SUA levels and nephrolithiasis in both males and females. A significant linear positive correlation between SUA levels and nephrolithiasis was shown when SUA levels increased to 356 µmol/L in men (*p* for nonlinearity = 0.1668), and the same linear positive correlation was shown when SUA levels increased to 265 µmol/L in women (*p* for nonlinearity = 0.0667). The OR of nephrolithiasis increased with increasing SUA levels in both males and females, and the trend of OR values with increasing SUA was similar (Fig. 4).

### Sensitivity analysis

The sensitivity analysis showed that the association between asymptomatic hyperuricemia and the risk of nephrolithiasis was robust. After adjusting for confounding factors, the OR with 95% CI was 2.109(1.862,2.388), *p* < 0.001 in model 1, 1.906(1.676,2.167), *p* < 0.001 in model 2 and model 3, and 1.457 (1.323–1.605), *p* < 0.001 in model 4, respectively. All ORs were slightly higher than the results of the main analysis (Table S1). After stratified analysis, hyperuricemia was significantly associated with an increased risk of nephrolithiasis in both males and females. The results of the restricted cubic spline analysis showed a dose–response association between SUA and the risk of nephrolithiasis prevalence in both males (*p* for nonlinearity = 0.7807) and females (*p* for nonlinearity = 0.0102), which was consistent with the results of the main analysis (Fig. S1).

## Discussion

In our cross-sectional survey of a multi-ethnic cohort from southwest China, hyperuricemia was independently associated with nephrolithiasis prevalence, and SUA showed a dose–response association with the risk of nephrolithiasis in both men and women, which persisted even after correction for possible confounders. This suggests that elevated SUA is an independent risk factor for nephrolithiasis even in an asymptomatic gout-free population and that this association is more stable in men.

Our main findings are consistent with previous studies. A study from Korea found that SUA levels were independently and moderately associated with an increased risk of nephrolithiasis in healthy adult men, and a dose–response relationship was observed in men, specifically a linear relationship between SUA reaching 300 µmol/L and the risk of developing nephrolithiasis [21], which is consistent with our view that elevated SUA is also an independent risk factor for nephrolithiasis. The difference is that in our study, a dose–response relationship between SUA levels and nephrolithiasis was observed not only in men but also in women, as shown by a linear positive association between SUA levels and the risk of nephrolithiasis in men after 356 µmol/L and in women after 265 µmol/L SUA levels. There are two main possible reasons for these differences. First, there were large differences in the gender and age composition of the participants in our study and that study. Previous studies have reported that the prevalence of hyperuricemia in women increases with age and that there is an age dependence of SUA levels in women [22, 23], and animal experiments have also found that estrogen may play a protective role in hyperuricemia by promoting renal clearance of SUA, which also supports the above view [24]. Secondly, Korea and China's geographical and human differences cannot be ignored. The occurrence of both hyperuricemia and nephrolithiasis is influenced by diet, lifestyle, dyslipidemia, and cardiovascular disease [13, 14, 25–27]. We considered the confounding effects of these factors while discussing the relationship of asymptomatic hyperuricemia with SUA levels and nephrolithiasis, so we also adjusted for demographic characteristics, lifestyle, and metabolism-related indicators and diseases, and the results were also robust after several tests.

In addition, our findings suggest some information on the prevention of nephrolithiasis. In the 2021 update of the expert consensus on the diagnosis and treatment of patients with hyperuricemia and high cardiovascular risk, several European investigators suggest defining hyperuricemia as an SUA level > 420 µmol/L in men and > 360 µmol/L in women [18]. The different prevalence of nephrolithiasis between men and women may be related to the different anatomical structure of urinary tract and the different rates of smoking and drinking between men and women. It is also possible that the discrepancy exists because androgens have the effect of increasing oxalate formation, whereas estrogen can increase the excretion of citric acid in the urine. The differences in the prevalence of nephrolithiasis among different ethnic groups may be due to differences in dietary habits among different ethnic groups, as well as different water quality in different regions. Also, the main sources of drinking water for residents in the same area vary, with three main sources: mountain springs, groundwater, and purified water, and confirming these conditions requires the use of more accurate measurement instruments and more sophisticated experimental designs. This suggests that we should add some more accurate measurements and tests of drinking water in community residents in the follow-up of the cohort. At the same time, we found some association between alcohol consumption and nephrolithiasis. Compared to study subjects who never drank alcohol, those who drank alcohol daily had a reduced risk of developing nephrolithiasis. This association was not observed in subjects who drank occasionally, 1–2 days per week and 3–5 days per week, which may be strongly related to the fact that we did not quantify alcohol intake, and further research is needed to investigate why daily drinkers in our study had a reduced risk of nephrolithiasis.

Asymptomatic hyperuricemia increases the risk of nephrolithiasis and there is an association between nephrolithiasis, hypertension, and hyperlipidemia. Pathophysiological and molecular biology-related studies have shown that uric acid (UA) can stimulate the production of reactive oxygen species (ROS) by vascular smooth muscle cells or uptake into endothelial cells via UA transport proteins, causing inflammation, oxidative stress (OS), and dephosphorylation of the endothelial-type nitric oxide synthase (eNOS), making nitric oxide (NO) bioavailability to decrease, thus inducing vascular endothelial dysfunction [28, 29]. Endothelial damage is an important step in the formation of nephrolithiasis [30, 31]. It can be assumed that inflammation, ROS production, and the development of OS played a role in the formation of nephrolithiasis [32, 33]. In addition, an association between SUA levels and microalbuminuria, a biomarker of endothelial dysfunction, had been found in human epidemiological studies [34, 35]. We will further investigate this association in the follow-up survey of our cohort study. Endothelial dysfunction as a newly identified systemic pathological condition characterized by the imbalance of all major endothelial mechanisms played an important role in the development of many metabolic diseases, including hypertension and hyperlipidemia [36–38]. It had been suggested that endothelial dysfunction is an intermediate clinical feature between nephrolithiasis and cardiovascular disease, and it had also been shown that nephrolithiasis as an influencing factor for hypertension [39]. This is consistent with the finding of a correlation between nephrolithiasis and hypertension and hyperlipidemia found in our study, but further studies are needed to elucidate the exact mechanism of their association.

There are some limitations of the present study that need to be mentioned. First, this is a cross-sectional study and our results do not allow us to determine the causal relationship between asymptomatic hyperuricemia and nephrolithiasis, and follow-up studies are needed to more accurately assess the relationship. Second, we used ultrasonography as the diagnostic method for nephrolithiasis, so we were unable to assess the type of nephrolithiasis. Although it is widely accepted that non-contrast enhanced computed tomography is the gold standard method for diagnosing nephrolithiasis disease, some studies suggest that ultrasound detection also has high sensitivity and specificity and is recommended for use during initial imaging and follow-up [40]. As a large sample community-based population study, the safety, feasibility, and economy of the diagnostic methods also need to be considered, so ultrasonography is more appropriate. In addition, the abdominal ultrasound examination of all subjects was performed by three fixed and experienced ultrasound doctors, and the rich clinical experience to some extent also helped to improve the detection rate in the study. Third, the information on the use of diuretics and uric acid-lowering drugs is lacking, both because CMEC was not a disease-specific cohort, but a cohort study focusing on the influencing factors of chronic disease in a community-based population in southwest China. Nevertheless, the strengths of our study include being based on a natural population, including a wide age range and a large sample size, and adjusting for some diets and lifestyles.

## Conclusions

In Chinese adults, hyperuricemia is associated with an increased risk of developing nephrolithiasis. When male SUA levels were higher than 356 µmol/L and female SUA levels were higher than 265 µmol/L, there was a dose–response relationship between elevated SUA levels and the risk of nephrolithiasis. In other words, before reaching the diagnostic criteria for hyperuricemia, the risk of nephrolithiasis rises with the increase in SUA. This suggests that controlling SUA levels may be significant for the prevention of nephrolithiasis.

### Supplementary Information

**Additional file 1: Table S1.** The association between hyperuricemia and nephrolithiasis after excluding the patients who self-reported nephrolithiasis in the sensitivity analysis. **Fig. S1.** Dose-response relationship between SUA and nephrolithiasis in males and females after excluding the patients who self-reported nephrolithiasis in the sensitivity analysis.

## Data Availability

The original contributions presented in the study are included in the article/supplementary material. The datasets used and/or analyzed during the current study are available from the corresponding author on reasonable request.

## Abbreviations

CKD: Chronic kidney disease
CMEC: China Multi-Ethnic Cohort
eNOS: Endothelial-type nitric oxide synthase
ESRD: End-stage renal disease
FBG: Fasting blood glucose
HDL-CH: High-density lipoprotein cholesterol
NO: Nitric oxide
OS: Oxidative stress
RBG: Random blood glucose
RCS: Restricted cubic spline
ROS: Reactive oxygen species
Scr: Serum Creatinine
SUA: Serum uric acid
TG: Cholesterol, Triglycerides
UA: Uric acid
