# Supplementary material for: Asymptomatic hyperuricemia associated with increased risk of nephrolithiasis: a cross-sectional study
Source: BMC Public Health. 2023 Aug 10;23:1525. doi: 10.1186/s12889-023-16469-y (PMC10416353; doi:10.1186/s12889-023-16469-y)
Supplement: Supplementary file 1 — Additional file 1: Table S1. The association between hyperuricemia and nephrolithiasis after excluding the patients who self-reported nephrolithiasis in the sensitivity analysis. Fig. S1. Dose-response relationship between SUA and nephrolithiasis in males and females after excluding the patients who self-reported nephrolithiasis in the sensitivity analysis. [file 12889_2023_16469_MOESM1_ESM.docx]

**Appendix**

We excluded patients who self-reported nephrolithiasis and redefined the nephrolithiasis patients as individuals diagnosed with nephrolithiasis through abdominal ultrasonography during the field survey. We adjusted for the covariates in a manner consistent with the main analysis, and the results are presented below.

**Multivariate binary logistic regression analysis**

After performing a multifactorial logistic regression analysis, with hyperuricemia as the only independent variable, we obtained an OR of 2.109 (95% CI: 1.862-2.388). Subsequently, we adjusted for participants' demographic characteristics, lifestyle behavior factors, and metabolism-related indicators and diseases in a sequential manner. We found that hyperuricemia remained a risk factor for nephrolithiasis, with ORs of 1.906 (95% CI: 1.676-2.167), 1.906 (95% CI: 1.676-2.167), and 1.762 (95% CI: 1.543-2.012). We also found that men had a higher risk of nephrolithiasis compared to women, and Bai’s people had a higher risk of nephrolithiasis than Han people; hypertension and hyperlipidemia were identified as risk factors for nephrolithiasis. No association was found between smoking, alcohol consumption, and nephrolithiasis **(Table S1)**.

**Restricted cubic splines regression**

The results of the restricted cubic spline analysis demonstrated a dose-response association between SUA and the risk of nephrolithiasis prevalence in both males and females. A significant linear positive correlation between SUA and nephrolithiasis was found in men (*p* for nonlinearity = 0.7180), and the nonlinear dose-response association was observed in women (*p* for nonlinearity = 0.0134). which was consistent with the results of the main analysis **(Fig. S1).**

**Table S1** The association between hyperuricemia and nephrolithiasis after excluding the patients who self-reported nephrolithiasis in the sensitivity analysis.

| Covariate | OR (95%CI) | | | |  |
| --- | --- | --- | --- | --- | --- |
|  | Model 0 | Model 1 | Model 2 | Model 3 |  |
| Hyperuricemia |  |  |  |  |  |
| No | Ref. | Ref. | Ref. | Ref. |  |
| Yes | 2.109(1.862,2.388)*** | 1.906(1.676,2.167)*** | 1.906(1.676,2.167)*** | 1.762(1.543,2.012)*** |  |
| Sex |  |  |  |  |  |
| Male |  | Ref. | Ref. | Ref. |  |
| Female |  | 0.543(0.487,0.606)*** | 0.543(0.487,0.606)*** | 0.545(0.489,0.608)*** |  |
| Ethnic Groups |  |  |  |  |  |
| Han nationality |  | Ref. | Ref. | Ref. |  |
| Yi nationality |  | 1.126(0.987,1.286) | 1.126(0.987,1.286) | 1.084(0.949,1.239) |  |
| Bai nationality |  | 1.360(1.198,1.545)*** | 1.360(1.198,1.545)*** | 1.332(1.173,1.514)*** |  |
| Hypertension |  |  |  |  |  |
| No |  |  |  | Ref. |  |
| Yes |  |  |  | 1.287(1.151,1.439)*** |  |
| Diabetes |  |  |  |  |  |
| No |  |  |  | Ref. |  |
| Yes |  |  |  | 1.123(1.002,1.259)* |  |

*Note:* Bolden numbers indicate statistical significance (**p* < 0.05, ***p* < 0.01, ****p* < 0.001)

Model 0: crude model (without adjustment);

Model 1: Model 0 adjusted for demographic features (i.e., age, sex, ethnic group, educational level);

Model 2: Model 1 adjusted for life behavior factors (i.e., smoking status, drinking frequency);

Model 3: Model 2 adjusted for metabolic-related indicators and diseases (i.e., BMI, FBG, Scr, Urea, hypertension, diabetes, fatty liver, hyperlipidemia).


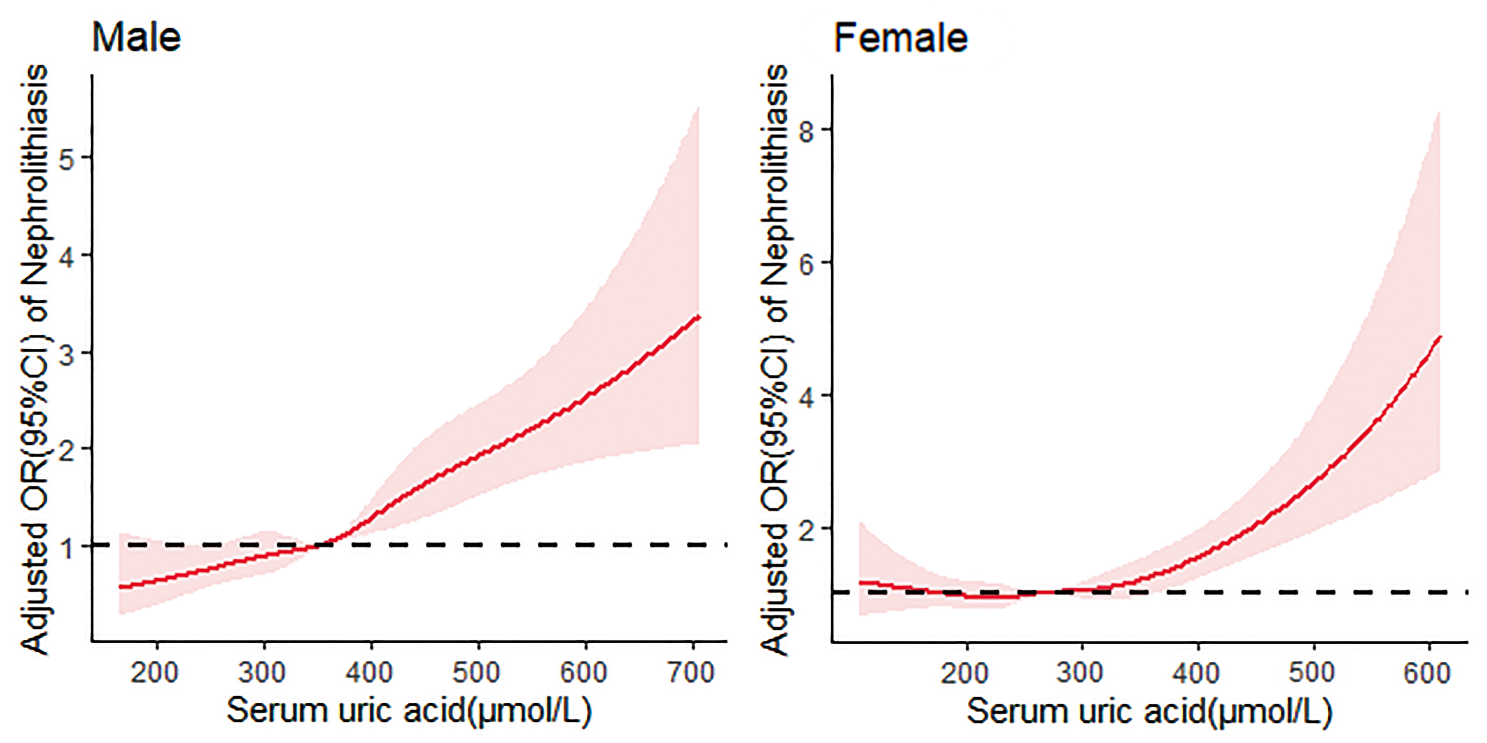


**Fig. S1** Dose-response relationship between SUA and nephrolithiasis in males and females after excluding the patients who self-reported nephrolithiasis in the sensitivity analysis.

Note: Adjusted for age, sex, ethnic groups, educational level, smoking status, alcohol drinking, BMI, fasting blood glucose, Scr, Urea, hypertension, diabetes, fatty liver, and hyperlipidemia.
